# Numerical Investigation on Progressive Collapse Mitigation of Steel Beam–Column Joint Using Steel Plates

**DOI:** 10.3390/ma15217628

**Published:** 2022-10-30

**Authors:** Mohammed Alrubaidi, S. A. Alhammadi

**Affiliations:** 1 Department of Civil Engineering, King Saud University, Riyadh 11451, Saudi Arabia; 2 Vice Rectorate for Facilities and Operation, Princess Nourah Bint Abdulrahman University, Riyadh 11564, Saudi Arabia

**Keywords:** upgrading, progressive collapse, shear joint, strengthening techniques

## Abstract

This research employed extensive numerical analyses to locate the weak areas and determine the structural issues critical to preventing the spread of collapse. As a result, three specimens were tested using scaled models of strengthened and unstrengthened steel beam–column joint assemblies. The data were utilized to verify numerical models. One simple shear joint from the three experimental assemblies was used as the control specimen (unstrengthened joint). The second was a bolted steel beam–column joint utilized as a reference specimen to reflect the ideal beam–column joint generally employed in intermediate moment-resisting frames in seismic zones worldwide. Similar to the control, the third specimen (strengthened joint) had two side plates welded together to strengthen the connection site. Numerical finite element models were developed using ABAQUS (2020) software to extensively investigate the behavior of steel frame assemblies before and after upgrading. The FEM matrix comprised 17 specimens with varying parameters, including plate thickness, steel grade, a joint between the beam flange-strengthening plates, and a column that was either welded or not welded. The effectiveness of the strengthening techniques was established by comparing the mode of failure and load–displacement characteristics of the investigated specimens. The results indicate that the average increase in peak load due to a change in plate thickness for grades A36 and A572 is approximately 22% and 8%, respectively. Plates made of A572 steel increase peak load by 30%. All strengthened specimens attained catenary action, mitigating the possibility of progressive collapse.

## 1. Introduction

Progressive collapse is defined as the spread of a local failure from one portion to the next, causing the structure or a substantial portion of it to collapse. An alternate pathway approach is used to resist structure collapse. In this strategy, gradual collapse does not occur if a crucial component (e.g., a column or foundation) fails. In case a column of steel building frame collapses due to blast or seismic loads, steel framed structures must have well-defined redundancies so that other load pathways are available by catenary action (CA) [1,2,3,4,5,6,7]. Thus, an upgrading technique for simple beam–column joints in steel frame buildings is necessary to mitigate the progressive collapse.

Many studies [3,7,8,9,10,11,12,13,14,15] have evaluated the increasing collapse risk of steel frame structures by removing one column and loading the frame at the column’s upper joint. This approach helps evaluate various load pathways and their effects on damage propagation to neighboring steel structural elements. Oosterhof, SA., and Driver, RG. [16] evaluated various types of simple joints under tension and shear-forces and found that the failure of shear steel joint specimens subjected to progressive collapse was governed by bolt tear-out failure mode and that the failure of bolted–bolted angle steel joint specimens under progressive collapse was controlled by tearing of the angle-heel section. Liu, C. et al. [17] studied the experimental and computational behaviors of two-span steel beams when the central steel column supporting them is immediately removed and determined that the experimental testing and the numerical curves were in excellent agreement.

Several different kinds of bolted steel beam–column connections were studied by Yang, B., and Tan KH. [18] using a middle column removal case. The bolted web angle steel joint fared best among the basic steel joints, whereas the double flange and web angle joints performed best among the semi-rigid steel joints in terms of load and rotation capacities. Li et al. [19] investigated a 1/3 scale single-story bare steel moment frame with welded steel beam–column joints subjected to a central-column-removal case and determined that the CA stage indicated a greater load was distributed to the strong axis columns than the weak axis columns. In this research, flexural action was shown to be the primary mechanism of collapse resistance in steel beam–column connections during the small displacement stage, whereas CA was the primary mechanism at the large displacement stage. Bae et al. [20] studied many probable scenarios for the gradual collapse of a steel-framed building. The removal of the corner columns was considered a primary cause of the building’s progressive collapse. Steel moment frames with stiffened beam–column connections were studied using a macro model by Han et al. [21], and their resistance to progressive collapse was determined. The bending of joints and the increase in catenary movement observed in actual progressive collapse may be captured by this model. For the progressive connection analysis, the researchers suggested a dynamic magnification factor of 1.6. On the topic of computational analysis, recent research that is relevant to this study has been conducted by Farzampour, A. [22], Paslar, N., and Farzampour, A. [23], and Farzampour, A. [24]. These studies are regarded as having a wealth of information in terms of computational analysis knowledge.

After exploring the literature on moment connections under column loss, Alrubaidi, M. et al. [6] provided an experimental and simulation analysis of the behavior of various intermediate moment resistant steel frame joints under column loss. The tensile resistance of the beam–column connections after substantial deformation was observed to typically influence the mechanism of failure and the development of catenary action. Zhong et al. [25] investigated the progressive collapse behavior of three steel building frame connections: welded unstrengthened flange-bolted web, top-seat angle with double web angle, and double web angle and showed that CA supplied most of the resistance of the double web angle specimen, whereas flexural action contributed more to the other two. The axial force at the top-seat angle with double angle joint loaded beam could be completely developed, resulting in superior column-loss resistance. Lew et al. [26] used a central-column-removal scenario to evaluate two types of bolted steel beam–column connections. The first connection had welded unstrengthened flange and fastened web, while the second had decreased beam section. The experimental findings for both connections under monotonic column movement were larger than the seismic test data. Wang et al. [27] examined the progressive collapse behavior of steel beams to concrete-filled steel tubular (CFST) column joints and showed that the steel frame with these connections could withstand progressive collapse after beam–column failure. In an intriguing work, Ozklc, YO., and Topkaya, C., [28] investigated experimentally and numerically the performance of extended unstiffened and stiffened end-plate connections utilized in replaceable shear links. According to the findings, end-plates constructed in accordance with AISC rules or Eurocode regulations demonstrate adequate performance in terms of the desired link rotation angle. Due to strain hardening effects, it was discovered that thinner plates than those specified by the regulations also demonstrated excellent performance. In their investigation, the authors also used finite element simulations to investigate the bending stresses for various plate thicknesses and the axial force levels created in the linkages. The authors recommended modifying the AISC design rules in order to more precisely determine the plastic resistance of end-plated connectors.

Steel beam–column joints must be successfully upgraded, which presented several difficulties. Previous published research literature on the subject of strengthening steel beam–column connections about mitigating or preventing progressive collapse have been lacking. For steel-framed buildings to improve their structural performance, some researchers, including Alrubaidi, M. et al. [29] (which is related to this work-study), have conducted experimental and numerical testing on two connection strengthening techniques. Even though the schemes generally reduced the risk of progressive collapse in steel frames, the study was limited because only two specimens met the criteria for each parameter. Analogous PT connections were studied by Christopoulos et al. [30], Rojas et al. [31], and Chou et al. [32]. Christopoulos et al. [30] investigated a connection using limited energy dissipating bars designed to yield in tension and compression, while Chou et al. [32] investigated a connection using steel plates yielding in tension and compression to provide energy dissipation. For the seismic retrofit of exceptional moment-resisting frames, Christopoulos et al. [33] presented self-centering structural solutions. The high-strength bars or tendons that produce the post-tension at each level make this a post-tensioned energy-dissipating (PTED) steel frame construction. Confining steel sleeves are often utilized to keep the energy-dissipating bars from buckling under the cyclic inelastic stress that occurs during seismic loading. Existing high-rise steel-framed structures might benefit from the novel retrofitting strategy by Liu, JL., [34], which entails transforming weak shear-resisting joints into robust moment-resisting joints. 

As explained previously, research on the strengthening of beam–column joints in steel-framed buildings to mitigate progressive collapse and the use of various parametric analyses has been lacking. The lack of such research makes investigations difficult. Thus, the purpose of this study is to analyze numerically the progressive collapse risk of steel beam–column joints reinforced with welded double side plates inside the connection area utilizing various schemes and parametric analyses. The design technique used to enhance the system was to provide continuity and redundancy in the load pathways in the case of column removal scenario. Twenty specimens were evaluated in this research, three of which were experimental: shear joint bolted joint and strengthened joint. These three provided a baseline against which the other 17 specimens were numerically tested using different parameters. Tests included a scenario the center column was lost, simulating a progressive collapse. The effectiveness of the strengthening techniques was investigated by comparing the results of the strengthened shear specimens to those of a reference unstrengthened shear specimen, and steel intermediate moment frame (IMF) joint specimen with bolted unstiffened extended endplate with pre-tensioned high-strength bolts designed and detailed as per ANSI/AISC 358-16 [35]. Three columns and two beams were used to construct the test frames.

The proposed strengthening system causes substantial improvement in the progressive collapse mitigation of the structure that is caused by the column damage, owing to the exposure of an extreme load event, leading to its ineffectiveness in carrying the loads. Although the proposed strengthening scheme is shown for a typical beam-–column connection, this can be easily implemented in all types of steel beam–column connections viz: (i) simple (pinned) connections, (ii) semi-rigid connections, and (iii) moment connections.

The importance of this system is of strengthening beam–column connections of steel frames for making them capable of developing catenary action in beams for resisting progressive collapse under extreme load scenarios such as blast-generated waves, vehicle crash against a column, or earthquake, the supported floors start caving in, which is resisted by the proposed strengthening scheme through the development of catenary action in beams. The proposed system is simple and easy to construct. The connections are made using commercially available steel plates. The scheme does not require very precise construction and fabrication tolerances. The FE models of the three steel assemblies with typical existing simple shear, moment and the strengthened specimen were validated in a previous study by Alrubaidi, M. et al. [29]. 

## 2. Experimental Specimens and Test Setup

**Figure 1:**
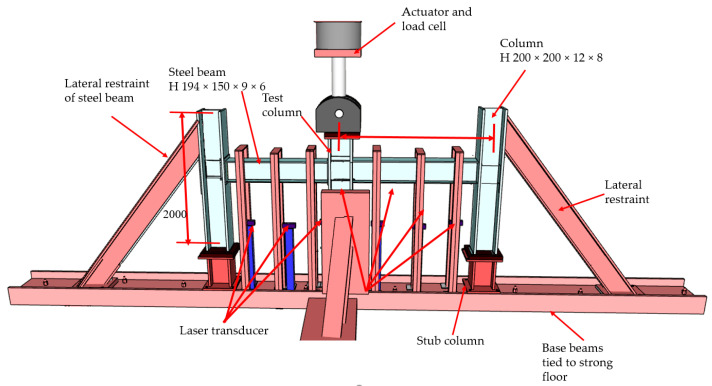
Details of test setup for steel frame.

**Figure 2:**
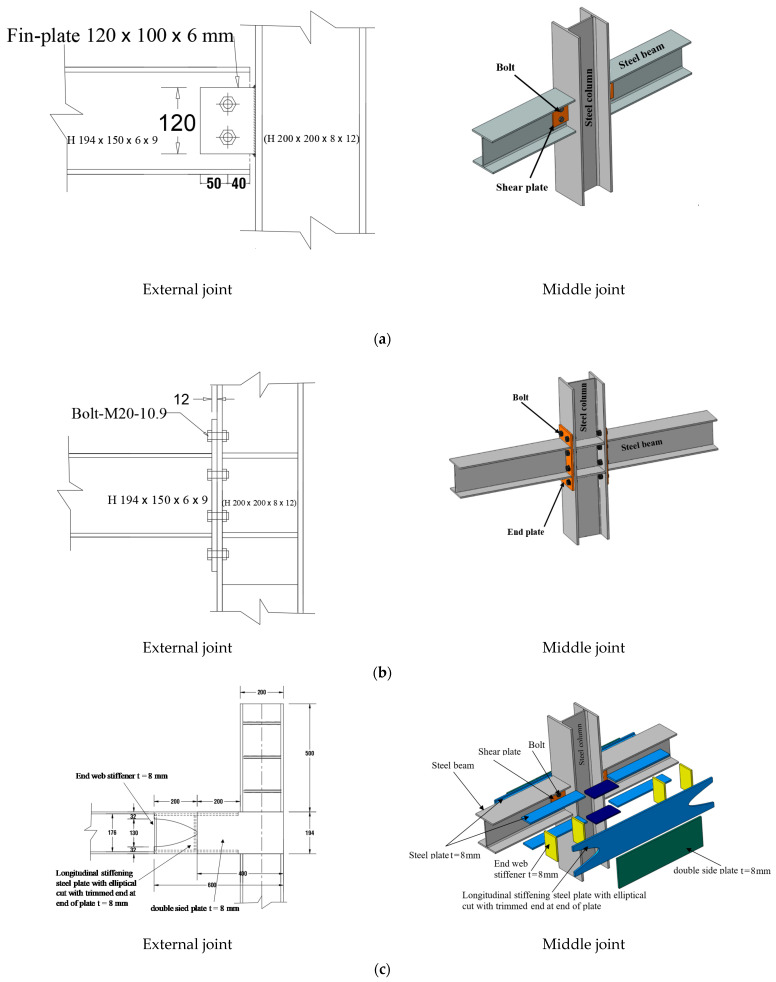
Details of external and middle steel beam–column joints: (**a**) Shear (simple) joint (J-S-C); (**b**) Details of IMF specimen (J-M-C); (**c**) Details of strengthened specimen (J-S-S-EX) [29].

An experimental study was completed at King Saud University to examine the progressive collapse resistance of steel beam-column assemblies having two types of simple shear connections under the loss of the middle column [6,29]. The purpose of this study is to compare the ability of strengthened and unstrengthened steel beam–column joints to mitigate progressive collapse should a ground-level column in the external frame be lost in a catastrophic event. The perimeter frames of the test specimens were created at a one-third size relative to the prototype. A total of three two-bay, two-dimensional steel frame assemblies were built, with specifications as shown in Table 1. Vertical loading tests were performed on specimens to gather data on the severe damage that would occur in a progressive collapse scenario. One such frame assembly is seen in Figure 1. The short column in the center represents the test steel column used to simulate the steel-column loss condition. The first of the three specimens (J-S-C) served as a control because it was built using a shear-type beam–column connection. The control specimen is a typical steel beam–column connection seen in countries without seismic activity. The second specimen (J-M-C) was designed to be an IMF beam–column connection; it consists of an extended end plate that is not stiffened and is connected to the column using high-strength bolts that have been pre-tensioned. The third specimen, J-S-S-EX, is designated as a strengthened specimen, which is similar to the shear control specimen J-S-C, but it was enhanced with welded double-side plates. All specimens have a span of 4 m with column spacing of 2 m and a total steel-column height of 2 m (Figure 1). Figure 2 depicts the steel beam–column connection details for the three test specimens. Table 1, Figure 1 and Figure 2 show that the rolled steel section H 194 was used in steel beams, and the rolled steel section H 200 was utilized in columns for all specimens.

A single-plate shear connection was used in the first J-S-C specimen, shown in Figure 2a. The web of the beam was fastened to the 6 mm shear plate with two M16 high-strength bolts of grade 10.9. The shear plate and column flange were welded together using a fillet weld. As illustrated in Figure 2b, the connection employed in the third specimen J-M-C was an IMF connection. The endplate bolted connection was made by welding an endplate onto the beam and then bolting the column flange onto the endplate using pre-tensioned high-strength bolts (M20-10.9). Following ANSI/AISC 358-16 [35], we directly tensioned all the bolts to a force of 179 kN. Figure 2c depicts the strengthening plan that was used for the J-S-S-EX specimen. The strengthening method shown in Figure 2c uses steel double plates joined at the internal beam–column connections of an existing steel frame. It is welded to the inner face of the lower and higher flanges of beams on each side of the column are eight beam flange-stiffening plates. Beam flange stiffeners and beam web stiffeners have their inner sides welded together, and so do eight-beam web stiffeners. In Figure 2c, we can see that the column web and the inner sides of the column flanges are strengthened by welding four column web stiffeners. The plates that strengthen the beam’s flanges and web are welded to two longitudinal stiffening plates with semi-elliptical cuts at both ends. Similar plates are frequently welded to the terminals of column web stiffeners. The ends of the longitudinal stiffening plates are removed for several reasons. First, the moment of inertia of the beam is not drastically altered by the cuts. Second, these holes enable inside-beam welding of the longitudinal stiffening plates to the beam web stiffening plates and beam flange stiffening plates. Additional steel plates are welded to the outer faces of the longitudinal stiffening plates halfway between the beam end and the column face. Beam ends are protected for a distance equivalent to 1.5 times their depth by the length of the connection that has been beefed up. For this reason, the steel double-plates strengthening scheme was developed with nominal material characteristics and no dynamic increase factor for strain rate effects. It was intended that the flexural and shear capacities of the beams in the enhanced specimen J-S-S-EX would be equal to those in the beams in the moment connection specimen J-M-C. Thus, the design was executed accordingly. The design was completed using ASTM A36 steel because it is widely available in the area. The thickness of each side and one plat was around 8 mm. 

**Figure 3:**
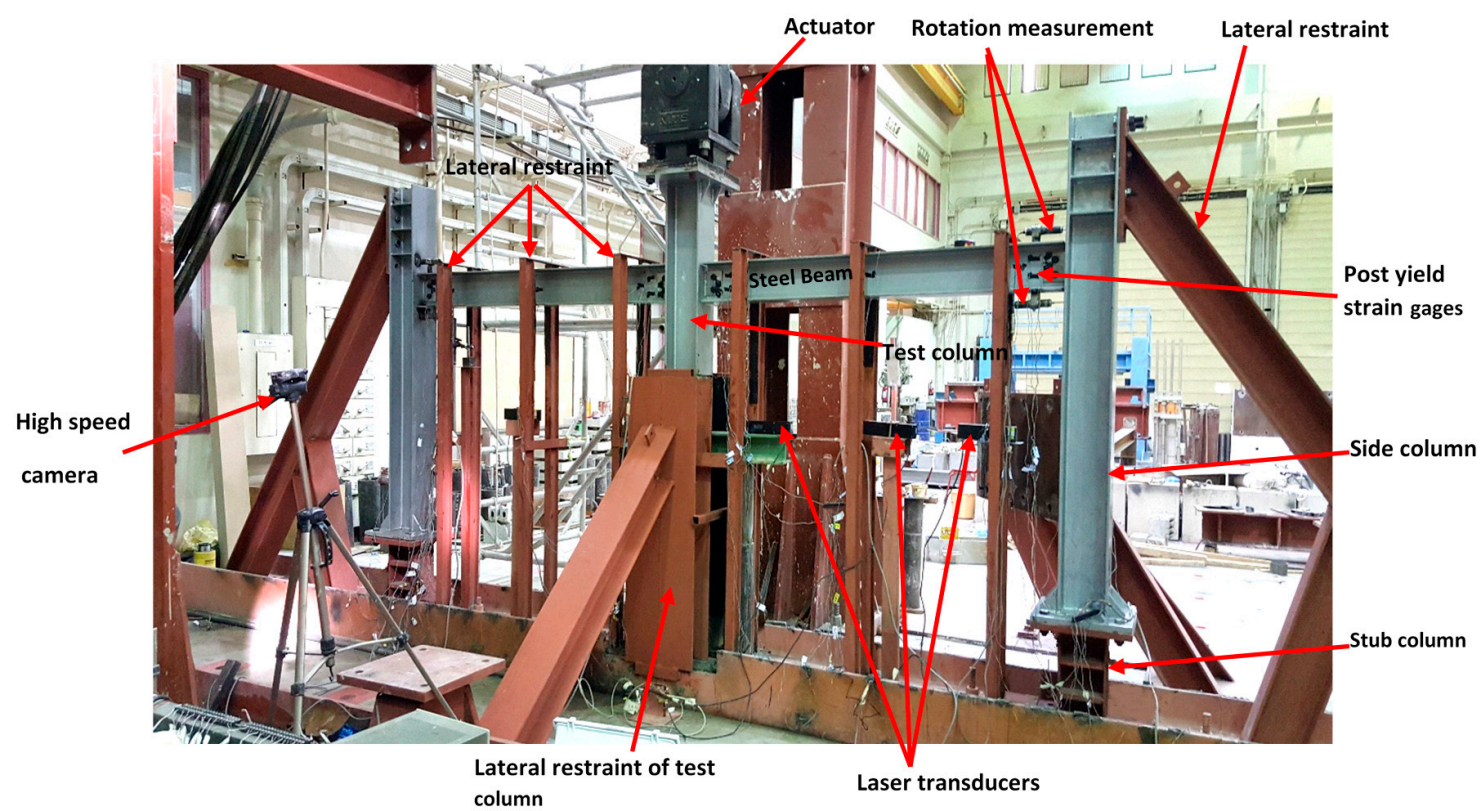
Instrumented specimen ready for testing [6].

The test set-up and equipment required the limiting of lateral out-of-plane movement of steel beams and excessive rotation at the end of columns for all specimens to acquire the behavior of the full-scale structure and duplicate the effect of surrounding stories. Figure 3 shows the lateral bracing system of the beam, which is done by partially extending the columns above beam level (with top ends constrained). A 1000 kN hydraulic high-speed actuator was loaded under servo control to simulate the removal of the central column of the steel frame. The actuator’s stroke length was 500 mm. The gradual collapse scenario was replicated by removing the support from the central column and applying a vertical force of 100 mm/s to the remaining column. Instruments were installed at important spots in the test frame to measure axial and torsional motions, as well as stresses induced by the connections. The displacements were measured using laser transducers stationed at different intervals along the beams. Under displacement control, the vertical displacement of the actuator was raised to apply progressively higher downward weights to the middle column. Stress measurements were possible because of the installation of post-yield strain gages at appropriate points on steel beam–column connections.

## 3. Experimental Results

Table 2 summarizes the findings of the experiments performed on the three tests used in this investigation. Key aspects of the load–displacement curve were determined through testing, including (i) load at a first yield of the steel-beam bottom flange on the joint area, (ii) maximum loads at the flexural and CA phases, (iii) center steel-column displacement at a first yield of the bottom flange of steel-beam at the inner joint, (iv) center steel-column displacements at peak loads at the flexural and CA phases, (v) center steel-column displacement at ultimate, (vi) peak axial force in steel-beam at CA phase, (vii) energy dissipated at maximum state, (viii) steel-beam rotation at peak load, and (ix) mode of failure. 

To calculate the axial load in the steel-beams N_u,CA_, we used strain gages to monitor the axial strain change in the steel-beam section along the beam depth, as shown in Figure 3. Next, the axial force of the steel beam was calculated by integrating the strain and normal stress distributions. To simplify, we assume that the rotations (θ) at both ends of the steel beam are similar. The flexural action resistance (P_u,FA_) and CA resistance (P_u,CA_) contribute equally to the overall resistance Pu [6,29]. The approximation of these factors may be found through the following:

*P_u,CA_* = 2 *N_u,CA_ sin θ*, (1)

*P_u,FA_* = *P_u_* − *P_u,CA_*. (2)

Peak vertical loads during the flexural and CA phases are shown in Table 2 for the test specimens and were calculated using Equations (1) and (2) [6]. The following is a summary of the test findings for each specimen.

**Figure 4:**
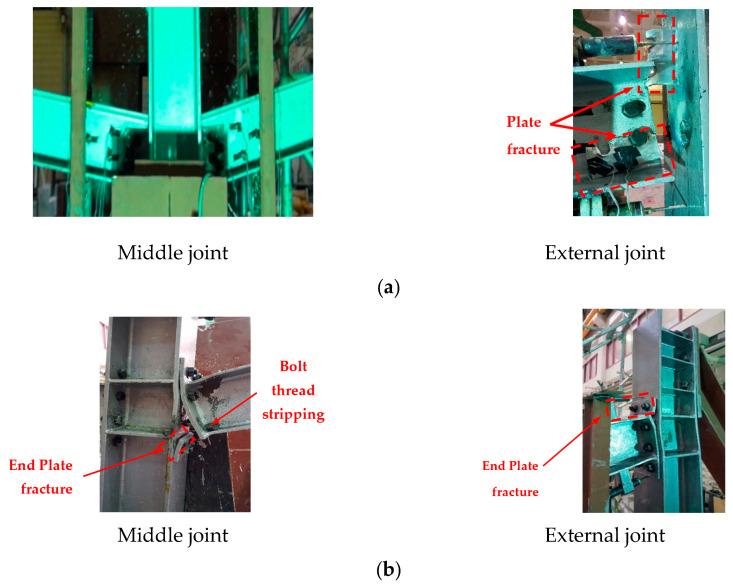

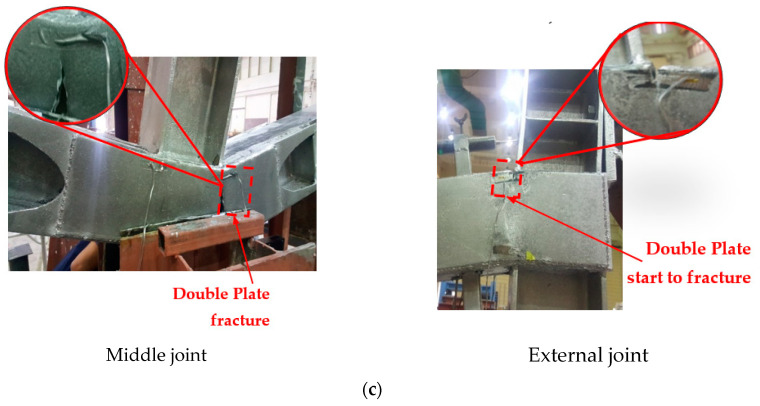
Failure-mode for experimental testes (external and middle joint): (**a**) J-S-C joint; (**b**) J-M-C joint; (**c**) J-S-S-EX joint [6,29].

**Figure 5:**
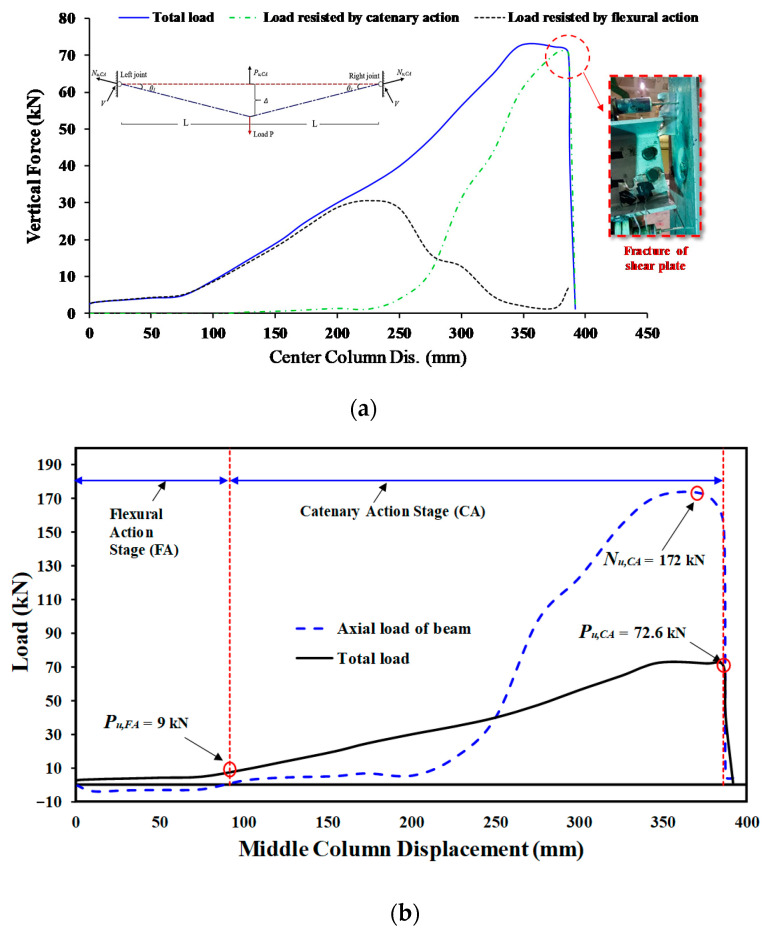
Characteristics of force and center steel-column displacement for J-S-C: (**a**) Curves of force and center column displacement, and (**b**) Curves of CA & flexural phases [6].

Figure 4a shows the observed mechanism of failure for J-S-C control specimen. Steel constructions with shear connections, as pictured, are subject to gradual collapse when a column fails under excessive load conditions. The rotation of the specimen at the two ends in response to an increase in the mid-span deflection is a frequent characteristic of such basic connections with poor moment-resisting capabilities. Figure 5 depicts the load–displacement characteristics of the J-S-C specimen. According to Figure 5, the specimen could tolerate low force up to a displacement of 75 mm. The axial force of the tensile steel beam was triggered at a displacement of 92 mm of the central steel column, indicating the onset of the CA behavior phase (Figure 5b). Bolt hole bearing deformations developed in the shear plate when the load was raised further. The whole steel frame failed at 72.6 kN when the shear tabs at the right joint fully broke (Figure 4a and Figure 5), where the steel column in the middle moved 386 mm.

**Figure 6:**
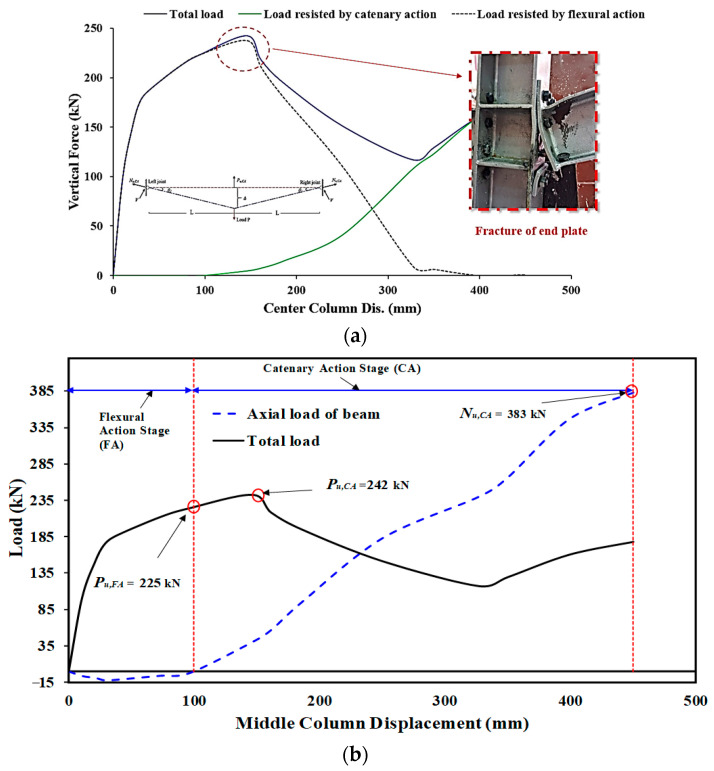
Characteristics of force and center steel-column displacement for J-M-C: (**a**) Curves of force and center column displacement, and (**b**) Curves of CA & flexural phases [6].

The test steel frame went through three different phases during the test of the J-M-C moment joint. The specimen was initially rigid because the bolts were pretensioned. However, after a 29 mm movement down for the middle steel column, the separation began at the middle connection between the column flange and the end plate, and the corresponding load was A 175 kN. Flexural stiffness decreased upon detachment (Figure 6), and as can be seen in Figure 4b, the end plate of the right beam at the middle joint began to bend. Until the end plate was bent, the axial force from the steel beam was very limited, as shown in Figure 6b. At a displacement of 146 mm, corresponding to a maximum applied stress of 242 kN, the right-hand beam’s end plate fractured at the middle steel column. After increasing the distance to 175 mm, the third step was the thread stripping failure of the bolts in the bottom second row (see Figure 4b). The axial force had little influence, and the flexure was the primary cause of the fractures. At a displacement of 250 mm, the endplates of both the left and right joints ruptured. Load increased as seen in Figure 6a with a further rise in displacement to about 350 mm. At this point, the bolts in the top left and right connections of the second row failed due to the extreme force being applied to them, and the threads on those bolts fell off.

**Figure 7:**
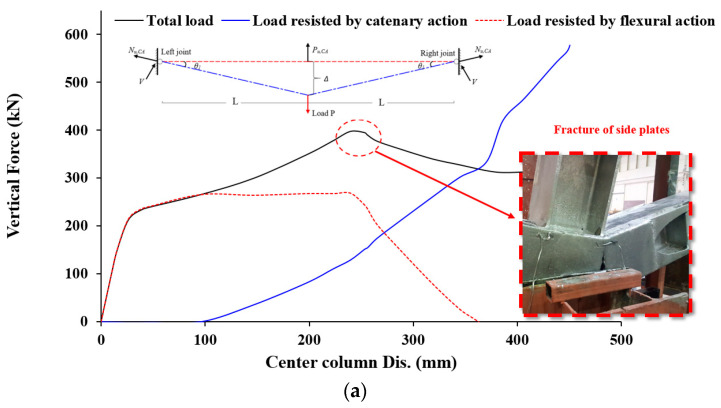

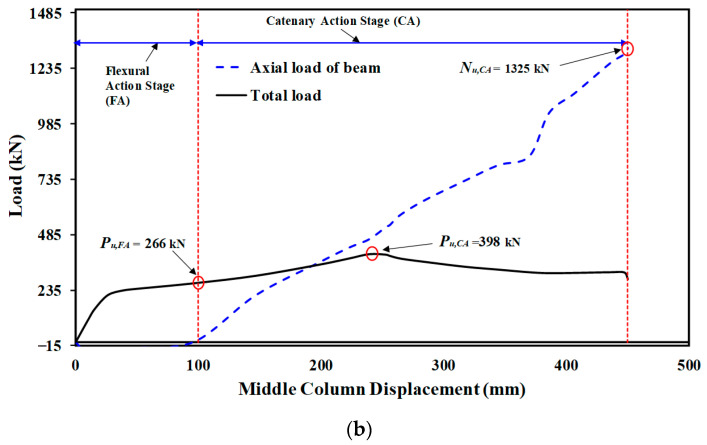
Characteristics of force and center steel-column displacement for J-S-S-EX (**a**) Curves of force and center column displacement, and (**b**) Curves of CA & flexural phases [29].

The failure mechanism of the J-S-S-EX specimen, which was strengthened with welded double-side steel plates within the joint area, is shown in Figure 4c. Figure 4c shows that the failure of the J-S-S-EX specimen began in the double side steel plates near the inner beam–column joint because of the combination of a fracture of the double side steel plates at the bottom edge and a fracture of the extreme plates. In Figure 4c, it can be observed that the fracture of the double-side steel plates began at the bottom edge and progressed to the top edge as a result of the propagation of flexural cracks in the maximum moment zone at the steel-column face. Figure 4c also shows that the upper edge of the plates bent somewhat on the top steel-beam side, close to the middle joint zone. Figure 4c further shows that the failure of the outer beam–column joint occurred much later in the test because of the fracture of double side plates. Load–displacement curves may be broken down into elastic, flexural, and catenary phases, with no sharp transition between them but rather interaction zones (Figure 7). Because the strengthened joint was rigid and no slippages were conceivable in the joint components, the specimen could withstand loads in the elastic range during the first loading phase, and the applied load grew virtually linearly. The onset of yielding was indicated by local buckling of the top edge of the right-side steel plates at the strengthened joint with the central steel column, and at this point, the displacement was 26 mm while the applied force reached 213 kN. It was at this point that the second phase began, whereby the flexural rigidity began to soften. During the pure flexural phase, the highest applied load was 266 kN, resulting in a vertical displacement of 97 mm. Steel beams were under compression (the axial force was negative) up to the 97 mm vertical displacement, with a maximum compression load of 25 kN (see Figure 7b), suggesting extremely little arching behavior in the structure. Most of the applied load was still resisted by the flexural capacity up to a vertical displacement of 100 mm, although the CA continued to increase after that. This phase, also known as the flexural-catenary phase, concludes with an applied force value of 266 kN (Figure 7b). The flexural resistance decreased after that, while the CA took center stage. The stiffness plateaued when the vertical displacement was 240 mm and applied force reached 398 kN. At this time, the high tensile force in the steel beams began to cause cracks in the end side plates of the right beam (near the center steel column), starting at the bottom side steel plates and progressing upwards and outwards. The amount of force needed to break the material decreased drastically as a result of the crack. Figure 7b shows the greatest axial force in steel beams, which was 1325 kN. The test was interrupted, and transducers were removed from the area around the center steel column because of how violently it cracked. At this stage, we measured 450 mm for the column’s center.

## 4. FE Modeling

A parametric numerical program that was developed using the ABAQUS (2020) software [36] is shown in this section. The parametric numerical program investigated the strengthening of steel beam–column joints using steel plates for progressive collapse mitigation using varying parameters, such as plate thickness, steel grade, a joint between the beam flange-strengthening plates, and a column that was either welded or not welded. In this study, the FE modeling software was based on the results of the experimental test’s calibration and validation. FE modeling involves five essential parts: material modeling, mesh creation, loading, and boundary conditions, FE model validation, and parametric analysis models.

### 4.1. Material Modeling

**Figure 8:**
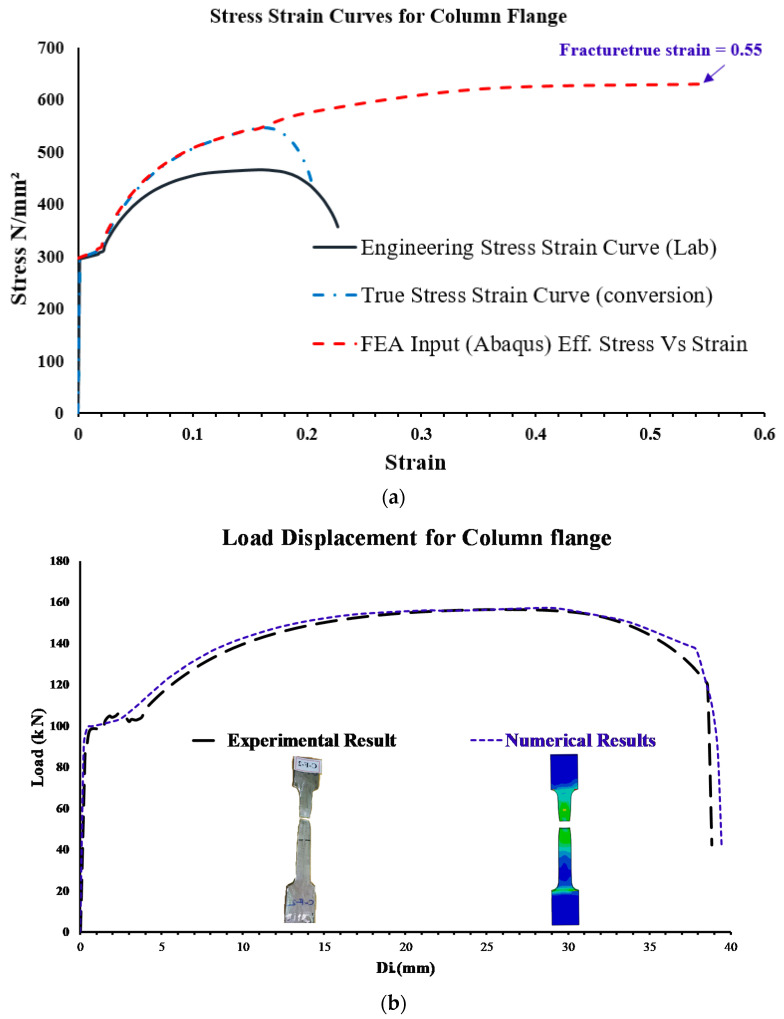

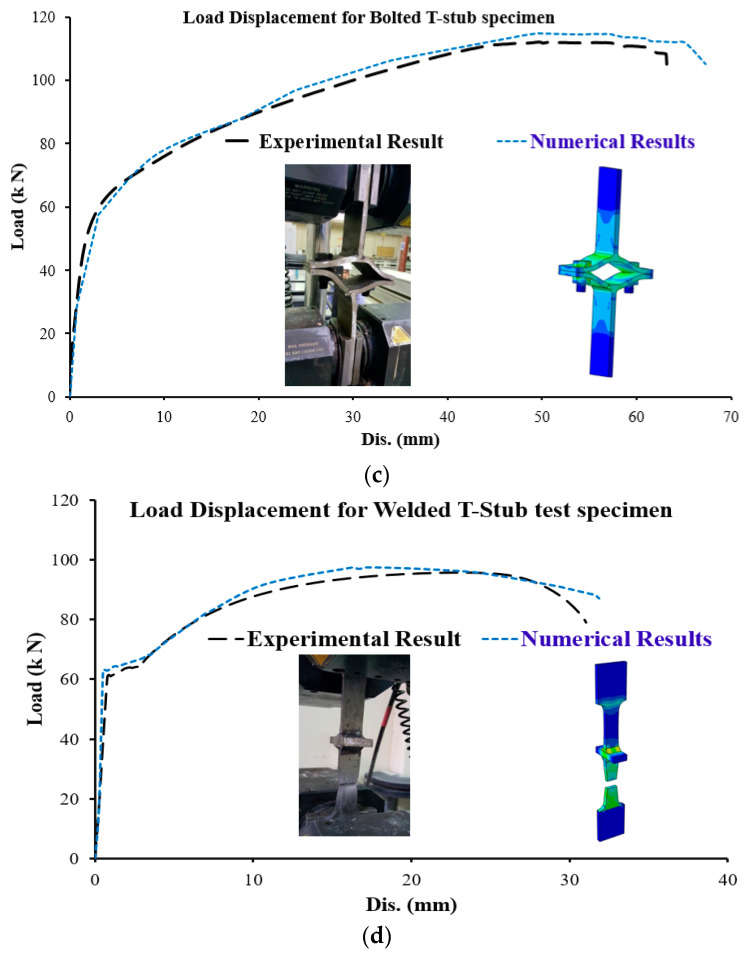
(**a**) Stress strain curves for the column flange coupon steel plate specimen, (**b**) Load versus displacement response for the column flange coupon steel plate specimen, (**c**) Load versus displacement response for the steel bolted T-stub specimen, (**d**) and Load versus displacement response for the steel welded T-stub specimen test.

Standard steel coupons (welded T-stub and bolted T-stub) were subjected to tensile testing in accordance with the 40. ASTM A370-05 [37] standard, and the input values utilized in the material model are shown in Table 3. By applying the following Equations (CEN, EN. “Eurocode 3, [38]) to the engineering stress–strain (σ_E_-ε_E_) curves obtained from each tensile test, we were able to get the true stress–strain (σtrue-εtrue) curves shown in Figure 8a.

(3) $$\sigma_{true}=\sigma_{E}\left(1+\varepsilon_{E}\right)$$

(4) $$\varepsilon_{true}=\mathrm{In}\left(1+\varepsilon_{E}\right)$$

Typical steel coupons appear to weaken after reaching their maximum load because to necking, although they are really hardening [6,29]. The true stress–strain figure (shown in Figure 8a) shows a rising parabolic curve after the engineering stress–strain relationship’s strength peak. To replicate the results of a tensile test on steel coupons, FE models in ABAQUS (2020) [36] were created using the actual dimensions of the coupon specimens. Iterative analysis was utilized to alter the element size of 8-nodded solid components. To establish a considerable agreement between the experimental and modeled load displacement curves for the three coupon types depicted in Figure 8, the FE mesh size was iterated. Elements were eliminated using ABAQUS’s “damage for ductile metal” [36] method to simulate the steel fracture found in the coupons during testing. This approach was used to remove elements from the FE model after finding the plastic fracture strain (Table 3) for different components using the true stress–strain curves presented in Figure 8a. Standard test samples of steel coupons, welded T-stubs, and bolted T-stubs have their experimental and FE failure modes compared in Figure 8. Good agreement was established between the experimental and FE results. This verifies that the element size and input material parameters are appropriate for simulating steel fracture by means of element erosion. Therefore, the 3D FE modeling of the steel frame assemblies, especially at beam–column connections, made use of the calibrated element size. 

It should be mentioned that in Figure 8a, only the column flange was utilized as an example, but comparable studies were carried out for other components of columns, beams, and plates, and three samples were obtained from each portion and the average result was taken.

### 4.2. Mesh Generation

**Figure 9:**
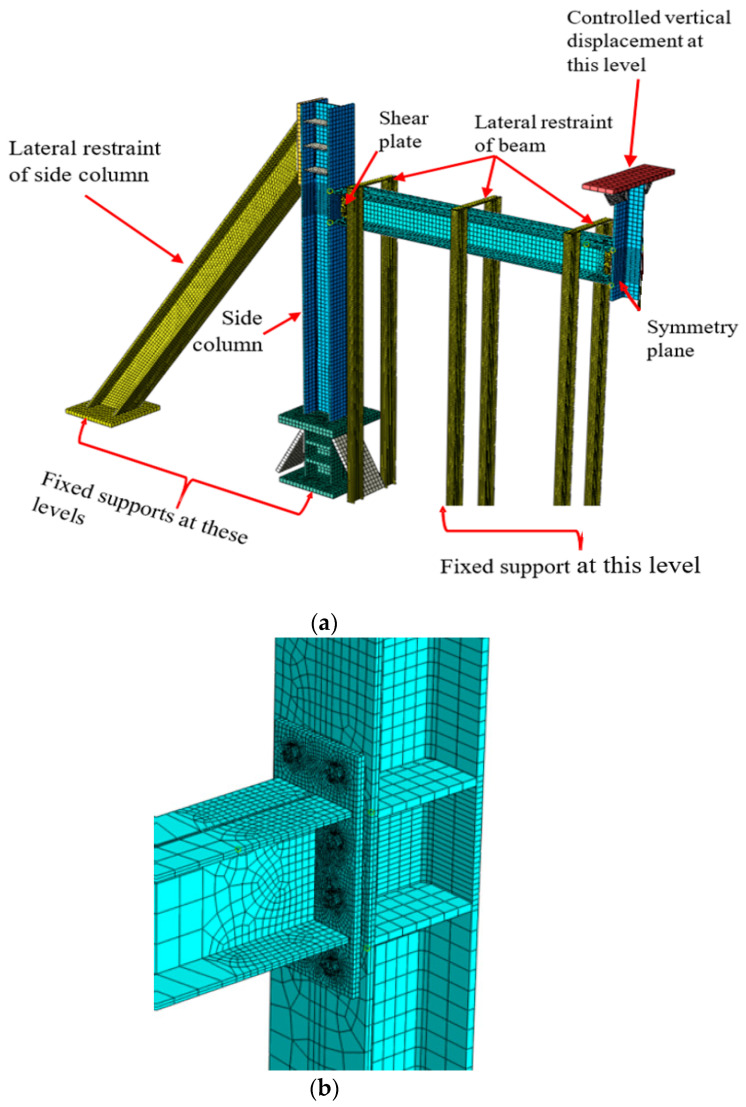
Finite element model for numerical investigation: (**a**) FE model for boundary conditions; (**b**) Details of joint elements.

Figure 9 provides an overview of the 2D test assembly’s entire numerical modeling. Multiple steel beams, steel columns, steel bolts, steel shear plates, and steel side plates were included in the FE mesh created using reduced integration of solid elements with eight nodes [6,29]. Solid elements ranging in size from 2 to 25 mm were employed in the FE simulation study. The steel bolts were modeled with a small mesh (2.0 mm), whereas the shear steel plates, steel columns, and steel beams had element sizes of approximately 5 mm at the ends of the steel beam where the shear steel plates ruptured (Figure 9b).

### 4.3. Loading and Boundary Conditions 

Figure 9a depicts how the boundary conditions of the FE used for the one-story 2D test assembly were very similarly matched to the boundary conditions of the test frames. The FEM followed the experiment’s lead by using the same lateral steel beam and column supports. In order to give more detailed modeling of the boundary conditions, the exact stiffness of the lateral bracing was represented using solid components. On the other hand, shorter columns were strongly bound to I-beams at the basement level, which were then attached to the laboratory’s concrete slab. Figure 9a demonstrates that as a result of this, the stub column bases were built to function as permanent supports. Out-of-plane movement of the steel beam flanges was prevented where lateral supports were installed. Bottom nodes prevented translation and rotation in all three dimensions (X, Y, and Z). All aspects of a building collapse were simulated using ABAQUS’ explicit module [6,29,36]. It is only necessary to simulate half of the specimens because the test frames are symmetric. Displacement developed at a rate of 100 mm/s, and the time history of the top nodes of the columns was set to match the displacement-controlled loads utilized in the tests.

### 4.4. FE Model Validation

The fidelity of the FE models was checked against experimental data collected for this study. The load–displacement characteristic and failure mechanism in the FE research results are reviewed in light of this validation. The primary load–displacement findings for the steel test frame are compared between experimental and numerical predictions in Table 2. Numerical modeling accurately anticipated all scenarios; therefore, only small variations were found between the FE findings and the test results.

**Figure 10:**
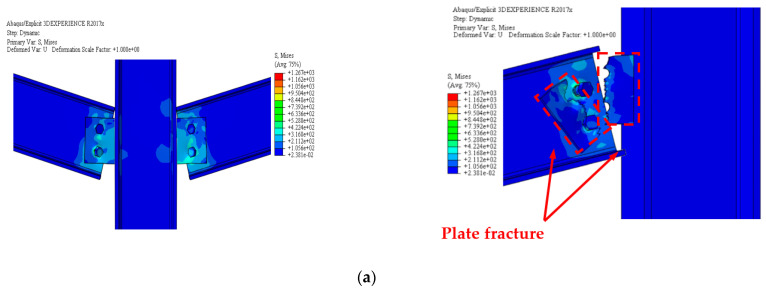

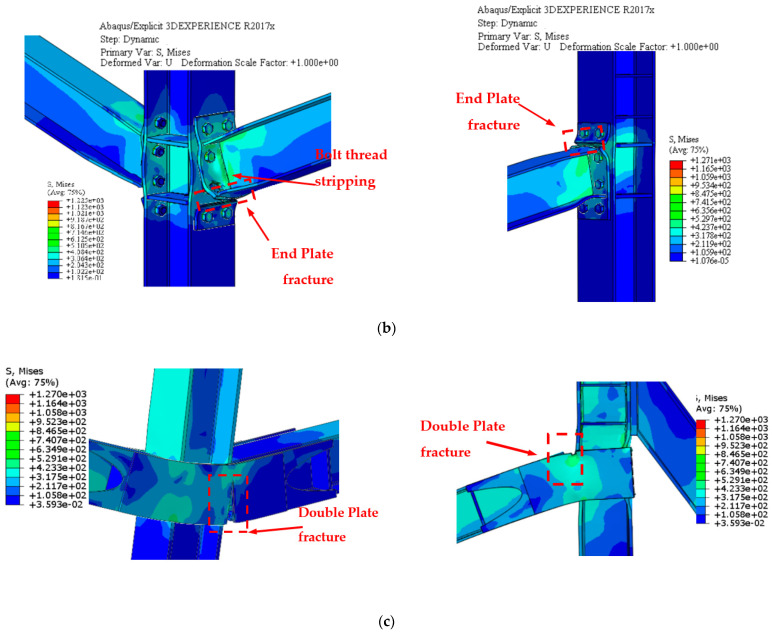
Failure-mode for FE mode testes (external and middle joint): (**a**) J-S-C joint; (**b**) J-M-C joint; (**c**) J-S-S-EX joint.

**Figure 11:**
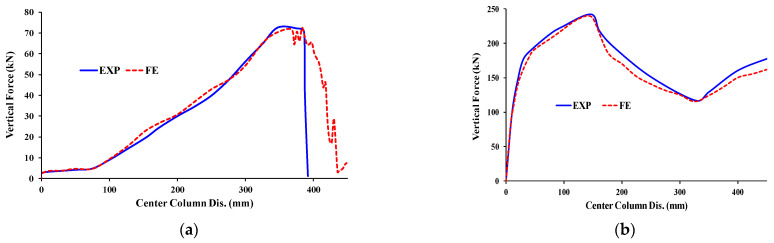

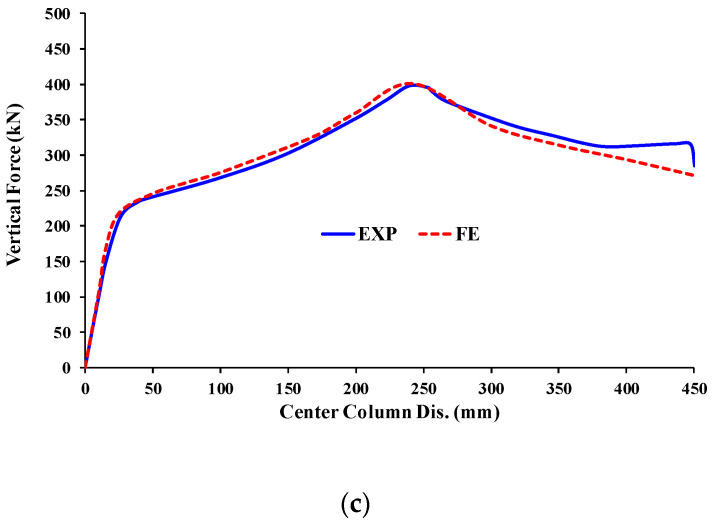
Curves of force and center column displacement comparison of experimental and FE for: (**a**) J-S-C; (**b**) J-M-C; (**c**) J-S-S-EX [29].

Figure 10 shows the numerically anticipated failure mechanisms of the three test steel frames are in good agreement with the data in the experimental. Figure 11 shows a visual representation of the calculated and experimental load versus the center column displacement for the two test frames. In addition, the experimental and numerically anticipated curves showed considerable consistency, especially at the highest load. Figure 11 shows both the computational and experimental evaluations of the test frames’ stiffness were conducted over the whole range of the responses. Figure 11 shows that the FE models, which used constitutive modeling, made accurate predictions of the load–displacement response’s falling component.

Calibration of all connections between the steel beam and the steel column, and how to carry it out under progressive collapse, was one of the validation objectives for the experimental specimens developed in this work. After finding agreement between experimental and numerical studies, the team decided to expand their investigation by conducting a parametric study, which considers the strengthening investigation of steel beam–column joint using steel plates for progressive collapse mitigation by varying parameters such as plate thickness, steel grade, a joint between the beam flange-strengthening plates, and a column that was either welded or not welded.

### 4.5. Models for the Parametric Analysis

**Figure 12:**
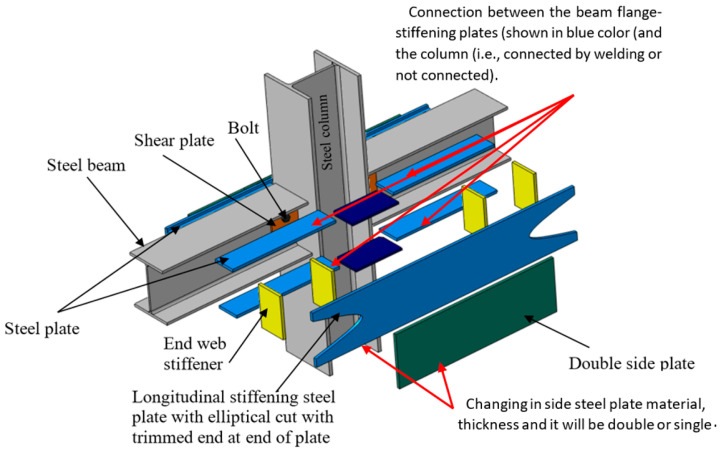
Details of strengthened joints used in the investigation study for J-S-S-EX.

The calibrated FEMs were expanded to examine the effect of varying side double steel plate scheme parameters on the behavior of upgraded steel frames caused by the elimination of the central column. The FEM matrix had 17 specimens with varied features, including steel plate thickness, steel grade (materials), and welding between the steel-beam flange-stiffening plates (shown in blue in Figure 12) and the steel-column (connected or not via welding) (see Table 4). The failure of specimen J-S-S-EX was attributed to the failure of the steel side plates, as shown by both experimental and FEM studies. As indicated in Table 4 and Figure 12, four steel plate thicknesses ranging from 8 to 15 mm were examined for ASTM A36 steel, while five plate thicknesses ranging from 6 to 15 mm were examined for ASTM A572 G50 steel. The quantitatively examined specimens had the same characteristics as the reinforced specimen J-S-S-EX shown in Figure 12.

## 5. FE Investigation of Different Parametric Study

### 5.1. Effects of Plate Thickness

**Figure 13:**
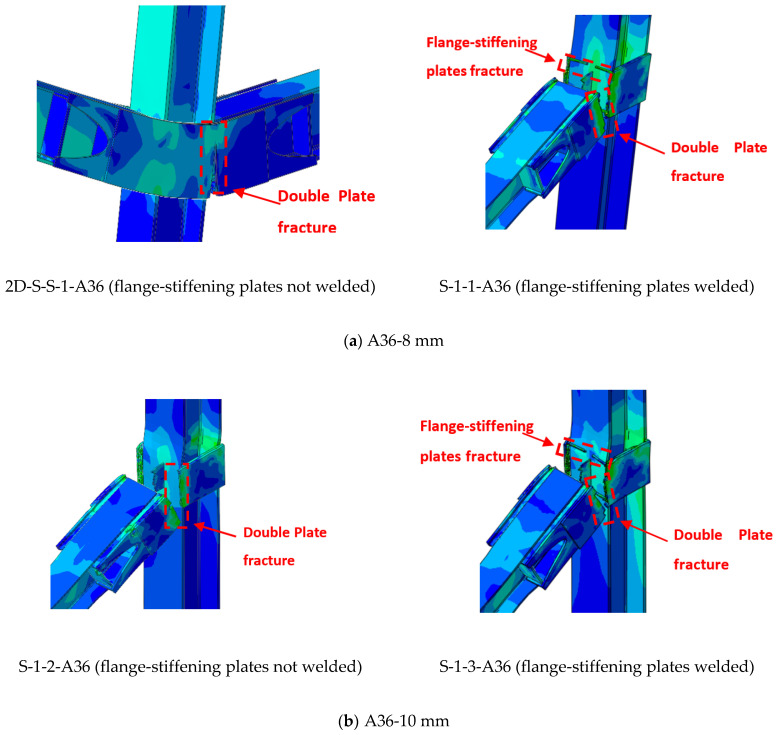

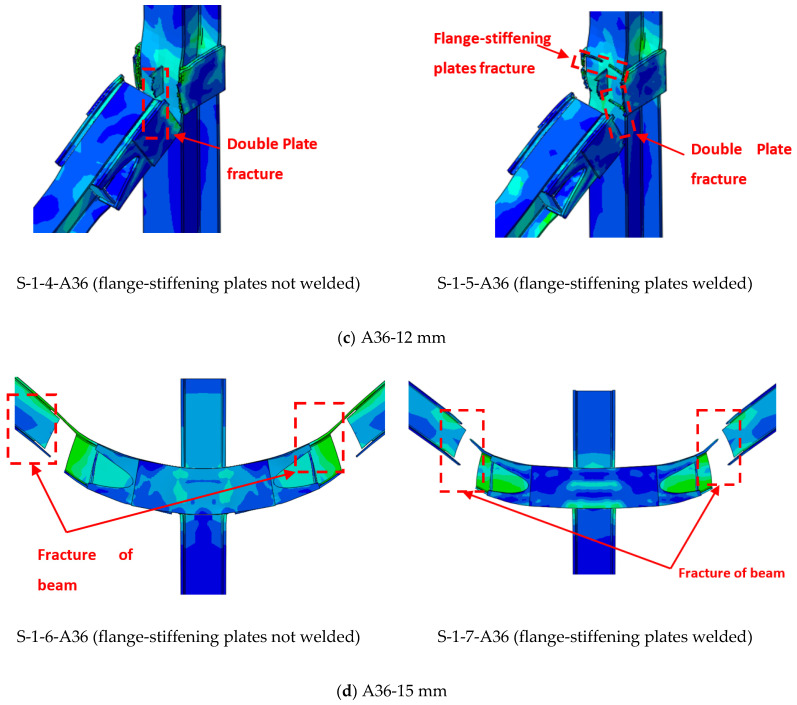
FEM mode of failure for strengthening scheme J-S-S-EXwith A36 materials.

**Figure 14:**
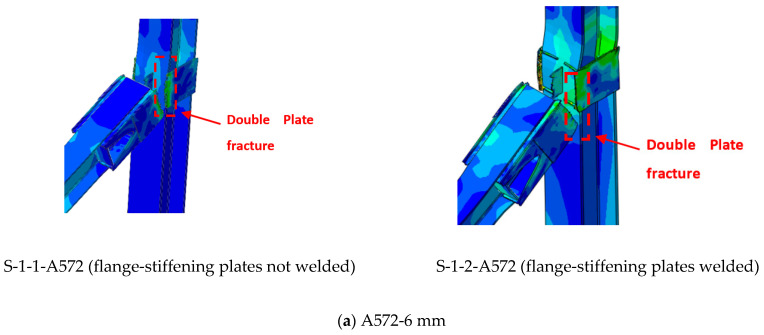

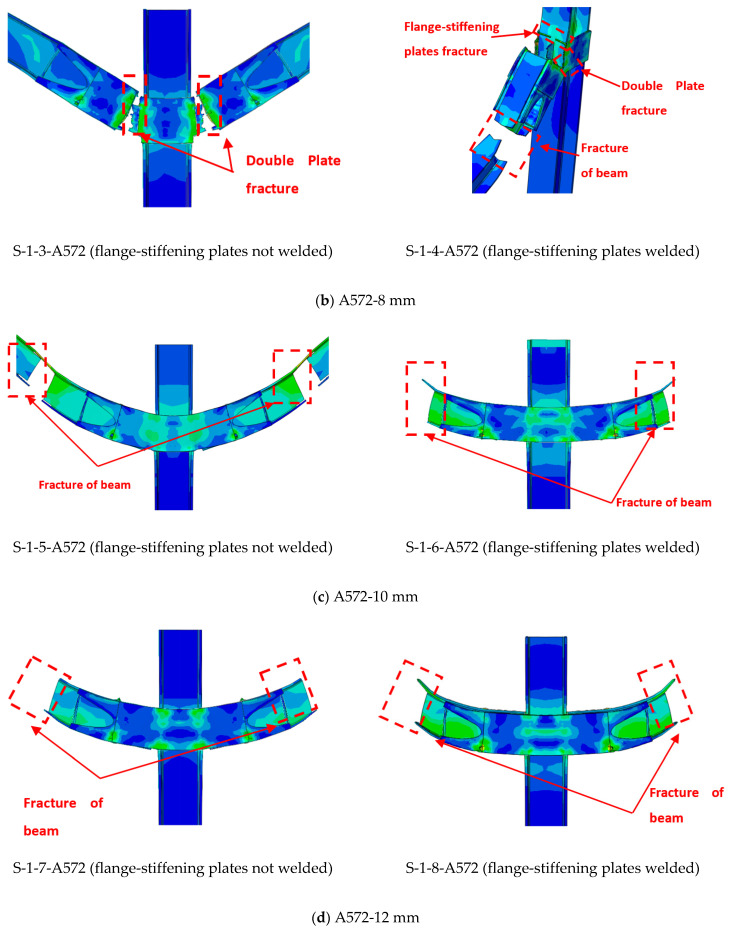

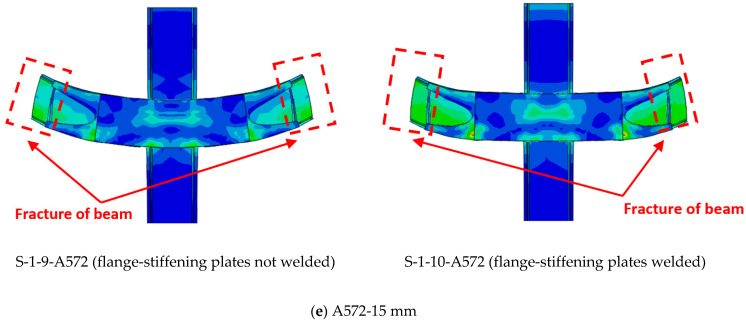
FEM mode of failure for strengthening scheme J-S-S-EX with A572 materials.

When considering the effect of plate thickness, other parameters are taken into account, such as the type of material, the welding, whether it is welded or not welded in the area of contact between the plates and the column, and whether the ring is from 6 mm to 15 mm thick. If we look carefully first, at the effect of thickness with the type of material (A36), we find the thickness from 6 mm to 12 mm, whether welded or non-welded, the collapse is in the critical region for sides plates, which is at the face of the column (Figure 13a–c). The ultimate mode of failure for specimens with thicknesses ranging from 6 to 12 mm (A36) and 6 to 8 mm (A572) occurs at the bottom edge of the double side plates surrounding the central connection, and it was coupled with severe plate fracture (Figure 13 and Figure 14). The fracture of the side plates then spread to the upper side of the beam. Later, at the end of the test, the outside connections failed because of the fracturing of the double side plates, as seen in Figure 13 and Figure 14. It is worth mentioning that the welded side plates split as a result of the usage of A36 steel plates ranging in thickness from 6 to 12 mm. The thickness of 15 mm in terms of collapse is in the case of the A36 materials, and whether welded or not welded to the face of the column, can transfer the collapse to the beam itself (Figure 13d).

**Figure 15:**
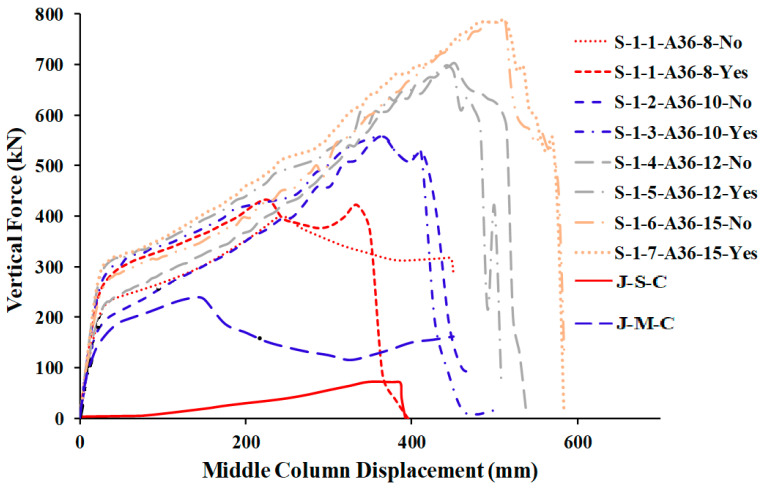
FEM force-displacement response for all A36 steel-specimens.

**Figure 16:**
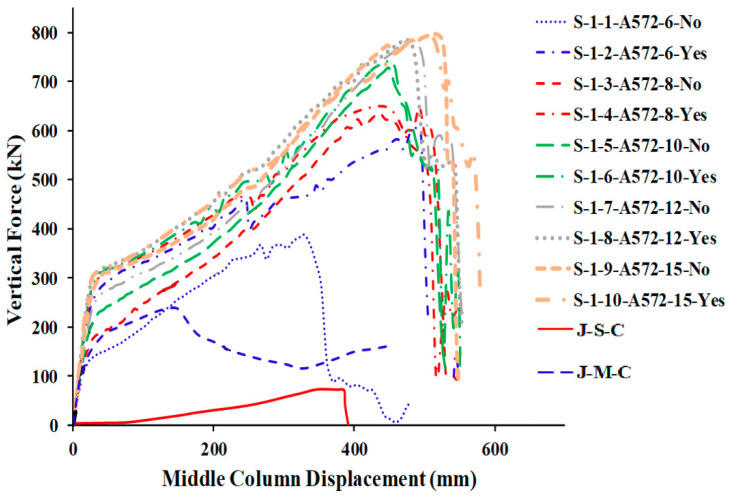
FEM force-displacement response for all A572 steel-specimens.

Table 5 shows the comparison of FEM results for welded specimens in terms of different plate thicknesses with two steel grades (A572 and A36) while Table 6 for not welded specimens. For the load and displacement side, it was found that all thicknesses play an excellent role in the beginning in terms of stiffness and is close for all thicknesses in this stage (Figure 15 and Figure 16). For the catenary action stage, the thicknesses of 12 mm and 15 mm are more effective in terms of load and displacement and is reflected in the energy dissipated, which helps to mitigate the progressive collapse in steel buildings when considering its strengthening using this technique with A36 materials, whether welded or not welded to the column.

### 5.2. Effects of Steel Grade (A36 & A572)

When considering the effect of materials type between A572 and A36, the change in materials certainly has a significant effect on raising the efficiency of joints in mitigating progressive collapse (Figure 13 and Figure 14), where it was found in the results in terms of the type of collapse that samples of type A36 with small thicknesses resist in the early stages, but the collapse occurs in the plates except for the thickness of 15 mm, the collapse occurs in the beam and this may be because of the thickness of the plate and not the type of material (Table 7). The A572 has a significant effect in mitigating progressive collapse, as the collapse began to turn into the beam with a thickness of 10 mm, indicating the effectiveness of the material A572 in contrast to the A36 (Figure 14). Concerning the load and displacement characteristics, samples with A572 material have good resistance at all stages, especially in the catenary action stage.

### 5.3. Effects of Welding of the Beam Flange-Stiffening Plates

To investigate the effects of welding the beam flange-stiffening plates to the removed center steel column on the progressive collapse (Figure 12), welding provides continuity in the contact area and has a positive effect on the process of resisting progressive collapse resulting from the removal of the column below this joint. However, welding, whether present or not in the contact area, depends entirely on the thickness of the plates, and most importantly, the type of materials (Table 5, Table 6 and Table 7). Welding with large thicknesses in A36 materials, especially 15 mm thickness, was effective in resisting progressive collapse, but slightly with the sample for the same thickness and without welding. Welding had a great effect on plates with A572 material, which may be due to the type of material and not the thickness or weld, because the difference is slight between welded and non-salt samples of the same thickness when the material is of the A572 type (Table 7).

## 6. Comparison of Parametric Study Results

**Figure 17:**
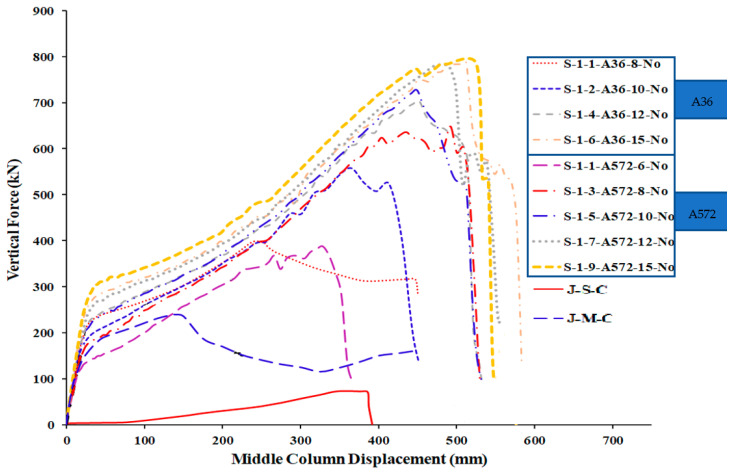
FEM force-displacement response for not welded A36 & A572 specimens.

**Figure 18:**
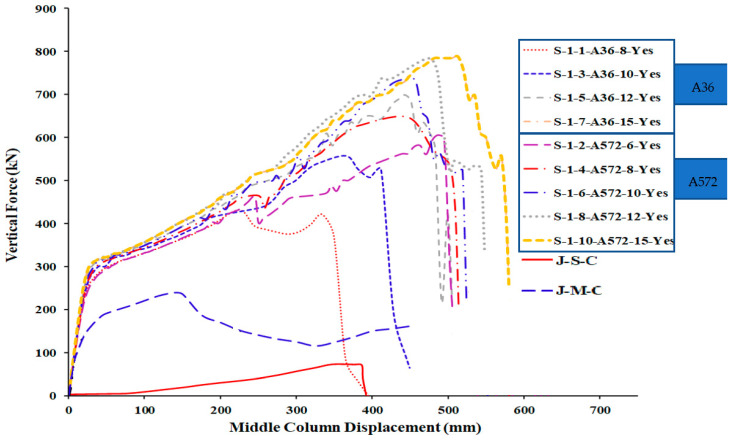
FEM force-displacement response for welded A36 & A572 specimens.

**Figure 19:**
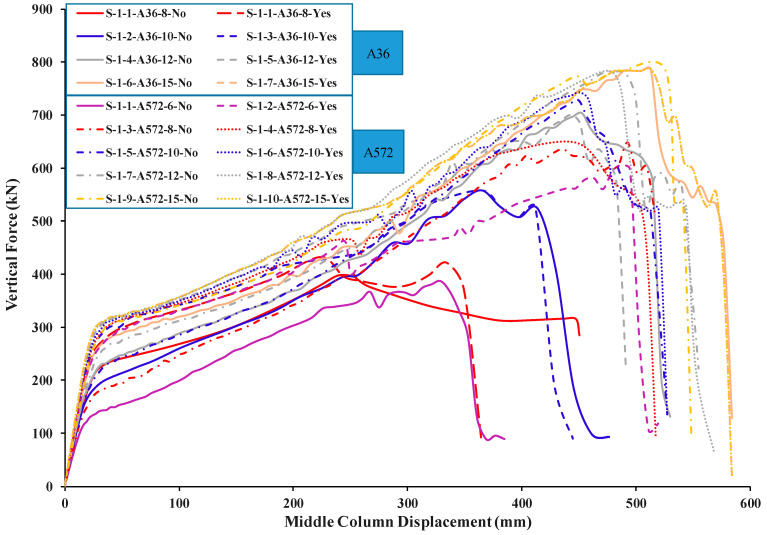
FEM force-displacement response for all FEM specimens.

The numerical outcomes of the 17 specimens used in the parametric research are shown in Table 5, Table 6 and Table 7. As shown in Figure 13 and Figure 16, the numerical mechanism failure for typical upgraded frames (with and without welding between the beam flange-stiffening plates and column flange) was the fracture of side steel plates at the middle column face. For the case of with and without welded beam, flange-stiffening steel plates with thicknesses of 10 and 15 mm for A572 steel and 15 mm for A36 steel (Figure 13 and Figure 14), flexural failure of the beam at the middle connection was predicted. For the case of welded beam flange-stiffening plates with a thickness of 8 mm for A572 steel, flexural failure of the steel beam at the exterior connection was predicted, as seen in Figure 14b. Load and displacement curves for representative specimens of studied steel frames are shown in Figure 15, Figure 16, Figure 17, Figure 18 and Figure 19. Specimens with either A36 or A572 steel plates displayed efficient load and displacement characteristics compared with simple shear and moment joints. Table 8 shows the outcomes from the parametric study of specimens. It should be noted that the increase in load carrying capacity due to welding between the beam flange-stiffening plates and column is about 25% (average) as seen in Table 8. Meanwhile, the increase in load due to the change in steel grade of plates from A36 to A572 is about 30% (average value) as seen in Table 5 and Table 7. The results of analysis for all of the parameters of this scheme show that the catenary action stage was reached in all specimens, which helps mitigate the progressive collapse, if not prevent it completely.

## 7. Recommendations and Future Research Directions

The following findings, which were derived from the research carried out for the study and offered as recommendations, are as follows:

- It was discovered that there is a limit to the progressive collapse resistance offered by simple beam–column connections. The linkages between these elements (beam and column) need to be strengthened so that the danger of progressive collapse may be reduced in significant existing buildings.
- It’s possible that the IMF connection, which is good for providing resistance to ground motions, will not be able to resist the progressive collapse.
- It is advised that a scheme be used for strengthening connections in multistory steel structures in order to mitigate the possibility of progressive collapse.
- The validated numerical models can be used for studying progressive collapse resistance of: (i) existing steel beam–column connections upgraded with any new strengthening scheme, (ii) newly developed steel moment beam–column connections.

As a result of the work that were carried out as part of this study, a number of possibilities and requirements relating to research have become apparent. The following is a list of significant areas that need additional investigation:

- Experimental and validated numerical finite modeling is recommended to study the behavior of different strengthening designs such as: post-tension wire systems within the beam–column connection.
- It is recommended to develop simplified reduced finite element models using beam and spring elements to predict the progressive collapse risk of different types of steel beam–column connections due to column-loss events. This may include simple shear, IMF, and strengthened connections, and the models will be validated using the experimental results of this study in addition to other tests available in the literature. The validated reduced models may be further utilized to study the progressive collapse risk of existing multistory steel framed buildings under column loss events. Additionally, the progressive collapse risk of the full steel framed buildings, simulated using reduced FEMs, may be numerically investigated under blast generated waves.
- Further research is needed to study experimentally and numerically the effect of composite RC slabs on reducing the progressive collapse potential of different types of steel beam–column connections (simple shear, IMF, special moment resisting frame, and strengthened connections) under column-loss scenarios. Effect of different designs of metal studs should be included.

## 8. Conclusions

The most important findings of the research are as follows:

- The shear-specimen J-S-C had a significantly high risk of progressive collapse because there were not enough load pathways available.
- The load-deformation response during a sudden column removal event was significantly enhanced by the employment of welded double-sided steel plates in the beam-to-column joint zone for the upgrading of shear beam-to-column joints. The enhanced frame J-S-S-EX could support around 5.5 times the maximum load of its predecessor, designated J-S-C (control specimen). However, in the final state, the amount of energy dissipated in the test frame J-S-S-EX was around 11.0 times that in the control frame J-S-C.
- The strengthened frame J-S-S-EX was able to support around 1.6 times as much weight as the IMF test frame J-M-C. In addition, the peak energy absorbed by test frame J-S-S-EX was almost 1.80% more than that of IMF specimen J-M-C.
- Three parameters were numerically investigated for strengthening of the shear beam–column connections using welded double side plates: (i) plate thickness, (ii) steel grade, and (iii) connection between the beam flange-stiffening plates and the column (i.e., connected by welding or not connected).
- It should be noted that the average increase in the peak load due to the change in plate thickness from 6 mm to 15 mm is about 22% and 8% for grades A36 and A572, respectively.
- The average increase in the peak load due to the change in steel grade of plates from A36 to A572 is about 30%.
- The average increase in the load-carrying capacity due to welding between the beam flange-stiffening plates and column is about 25%. The results of the analysis for all parameters of this scheme show that the catenary action stage was reached in all specimens, which helps mitigate (or diminish) the progressive collapse risk.
- The specimen that used a side plate with 15 mm of thickness, regardless of whether it was made of A36 or A572 material, showed the greatest performance in load displacement characteristics when subjected to the progressive collapse test out of all of the FE tested specimens.

## Figures and Tables

**Table 1:** Details of the tested steel frame specimens [29].

ID of Specimen	Connection Type	Size of Steel Plate (mm)	Side Plates t (mm)	I-Section Column (mm)	I-Section Beam (mm)	Bolt Grade 10.9
J-S-C	Simple (Shear)	shear steel plate 120 × 100 × 6	-	H × B × t_f_ × t_w_ 200 × 200 × 12 × 8	H × B × t_f_ × t_w_ 194 × 150 × 9 × 6	D16 (mm)
J-M-C	IMF	shear steel plate 120 × 100 × 6	-	200 × 200 × 12 × 8	194 × 150 × 9 × 6	D 20 (mm)
J-S-S-EX	Strengthened specimen	-	6	200 × 200 × 12 × 8	194 × 150 × 9 × 6	D16 (mm)

**Table 2:** Evaluating the force displacement response of experiments and finite elements [29].

ID Specimen	Type of Results	Flexural Phase				Catenary-Action Phase				Progressive Collapse Resistance Pu (kN)	Displacement at Ultimate State Δu (mm)	Energy Dissipated at Ultimate State Eu (kN.m)	Beam Rotation at Maximum Load θ (degree)	Failure Mode
		Yield Load-Py (kN)	Displacement Δy (mm)	Peak Load of Flexural Pu, FA (kN)	Displacement of Flexural Δu,c-FA (mm)	Peak Load of Catenary Action Pu, CA (kN)	Pu, CA/Pu, FA	Displacement of Catenary Action Δu,c-CA (mm)	Peak Beam Axial force Nu, CA (kN)					
J-S-C	EXP	-	-	9	92	72.6	8.07	375	172	72.6	386	12.5	11.9	Fracture of tab plate
	FE	-	-	9	92	71.5	7.94	383	172	71.5	383	12.7	11.8	
	EXP/FE	-	-	1.00	1.00	1.02	1.02	0.98	1.00	1.02	1.01	0.98	1.01	
J-M-C	EXP	138	20	225	100	242	1.08	146	383	242	187	78.0	3.9	Fracture of end plate
	FE	138	20	223	100	240	1.08	139	373	240	173	73.5	3.7	
	EXP/FE	1.00	1.00	1.01	1.00	1.01	1.00	1.05	1.03	1.01	1.08	1.06	1.05	
J-S-S-EX	EXP	213	26	266	97	398	1.5	240	1325	398	450	138	8.0	Fracture of double side plate
	FE	218	25	275	100	401	1.46	242	1335	401	450	139	8.1	
	EXP/FE	0.98	1.04	0.97	0.97	0.99	1.03	0.99	0.99	0.99	1.00	0.99	0.99	

**Table 3:** Material properties of different components of tested specimens.

Components	Elastic Modulus E (GPa)	Yield Strength f_y_ (MPa)	Tensile Strength f_u_ (MPa)	Elongation at Max. Stress δ_ms_ (%)	Elongation at Fracture δ_f_ (%)	Fracture True Strain
Column steel-flange	204	294	467	17	21	0.55
Beam steel-flange	193	330	470	16	22	0.60
Column steel-web	211	303	463	15	21	0.56
Beam steel-web	195	361	470	16	18	0.61
End steel-plat	193	281	432	13	19	0.60
Shear steel-plate	193	282	355	15	22	0.60
Side steel-plate	195	219	347	16	27	0.62
Bolt class 10.9	211	958	1070	-	10.2	0.21

**Table 4:** Details of strengthened joints used in the investigation study for J-S-S-EX.

Specimen ID	Parametric Study for J-S-S-EX			Details
	*t_p_* (mm)	Grade	Beam Flange-Stiffening Plates Welded to Flanges of Column	
Grade of side steel plate material (A36 (Fy = 220 MPa)				All specimens (parametric study) in this Table are same as J-S-S-EX but with changing in: Side steel plate thickness. Grade of side steel plate material (A36 (F_y_ = 220 MPa) or A572 (F_y_ = 408 MPa)). Connection between the beam flange-stiffening plates (shown in blue color (see Figure 12) (and the column (i.e., connected by welding or not connected).
S-1-1-A36	8	A36	YES	
S-1-2-A36	10	A36	NO	
S-1-3-A36	10	A36	YES	
S-1-4-A36	12	A36	NO	
S-1-5-A36	12	A36	YES	
S-1-6- A36	15	A36	NO	
S-1-7- A36	15	A36	YES	
Grade of side steel plate material (A572 (Fy = 408 MPa)				
S-1-1-A572	6	A572	NO	
S-1-2-A572	6	A572	YES	
S-1-3-A572	8	A572	NO	
S-1-4-A572	8	A572	YES	
S-1-5-A572	10	A572	NO	
S-1-6-A572	10	A572	YES	
S-1-7-A572	12	A572	NO	
S-1-8-A572	12	A572	YES	
S-1-9-A572	15	A572	NO	
S-1-10-A572	15	A572	YES	

**Table 5:** Comparison of FEM results for welded specimens in terms of different plate thicknesses with two steel grades (A572 & A36).

Specimen ID (Plate Thickness)	Materials	Load of Flexural Action Stage (kN)	Load at Ultimate State (kN)	Mode of Failure
6 mm-welded	A572	400	605	Fracture of side plates
	A36	-	-	Fracture of side plates
	(A572/A36)	-	-	-
8 mm-welded	A572	426	648	Beam failure
	A36	414	431	Fractures in the side plates
	(A572/A36)	1.03	1.50	-
10 mm-welded	A572	444	742	Beam failure
	A36	426	557	Fractures in the side plates
	(A572/A36)	1.04	1.33	-
12 mm-welded	A572	447	784	Beam failure
	A36	448	699	Fractures in the side plates
	(A572/A36)	1.00	1.12	-
15 mm-welded	A572	457	787	Fracture of beam
	A36	465	787	Fracture of beam
	(A572/A36)	0.98	1.00	-

**Table 6:** Comparison of FEM results for not welded specimens in terms of different plate thicknesses with two steel grades (A572 & A36).

Specimen ID (Plate Thickness)	Materials	Load of Flexural Action Stage (kN)	Load at Ultimate State (kN)	Mode of Failure
6 mm-not welded	A572	301	387	Fracture of side plates
	A36	-	-	Fracture of side plates
	(A572/A36)	-	-	-
8 mm-not welded	A572	338	648	Fracture of side plates
	A36	349	400	Fracture of side plates
	(A572/A36)	0.97	1.62	-
10 mm-not welded	A572	368	727	Beam failure
	A36	357	557	Fractures in the side plates
	(A572/A36)	1.03	1.33	-
12 mm-not welded	A572	391	783	Beam failure
	A36	372	702	Fractures in the side plates
	(A572/A36)	1.05	1.11	-
15 mm-not welded	A572	416	784	Fracture of beam
	A36	397	787	Fracture of beam
	A572/A36	1.05	1.00	-

**Table 7:** Comparison of FEM findings from specimens used in the investigation study.

Specimen ID	Welded	Load of Flexural Action Stage (kN)	Load at Ultimate State (kN)	Mode of Failure
Grade of side steel plate material (A36 (Fy = 220 MPa)				
A36-8 mm	Yes	414	431	Fracture of side plates
	No	349	400	Fracture of side plates
	Yes/No	1.18	1.07	-
A36-10 mm	Yes	426	557	Fracture of side plates
	No	357	557	Fracture of side plates
	Yes/No	1.19	1.00	-
A36-12 mm	Yes	448	699	Fracture of side plates
	No	372	702	Fracture of side plates
	Yes/No	1.20	0.99	-
A36-15 mm	Yes	465	787	Fracture of beam
	No	397	787	Fracture of beam
	Yes/No	1.17	1.00	-
Grade of side steel plate material (A572 (Fy = 408 MPa)				
A572-6 mm	Yes	400	605	Fracture of side plates
	No	301	387	Fracture of side plates
	Yes/No	1.33	1.56	-
A572-8 mm	Yes	426	648	Fracture of beam
	No	338	648	Fracture of side plates
	Yes/No	1.26	1.00	-
A572-10 mm	Yes	444	742	Fracture of beam
	No	368	727	Fracture of beam
	Yes/No	1.21	1.02	-
A572-12 mm	Yes	447	784	Fracture of beam
	No	391	783	Fracture of beam
	Yes/No	1.14	1.00	-
A572-15 mm	Yes	457	787	Fracture of beam
	No	416	784	Fracture of beam
	Yes/No	1.1	1.00	-

**Table 8:** Outcomes from parametric study of (J-S-S-EX).

Parametric Study	Average Increase in the Peak Load	Mode of Failure
Plate thickness	From 6 mm to 15 mm is about 22%	Shifting the failure from connection to beam
Steel grade (A36 & A572)	From A36 to A572 is about 30%	Fracture of double side plate (A36) Shifting the failure from connection to beam (A572)
Weld the beam flange-stiffening plates	From not welded to welded is about 25%	Fracture of double side plate (not welded) Shifting the failure from connection to beam (welded)

## Data Availability

Not applicable.
